# The Past Year and the Future

**Published:** 1912-08

**Authors:** 


### BUFFALO MEDICAL JOURNAL

**A Monthly Review of Medicine and Surgery**

*EDITOR*

A. L. BENEDICT.

All communications, whether of a literary or business nature, books for review and exchanges, should be addressed to the editor, 354 FRANKLIN STREET, BUFFALO, N. Y Please make personal or telephonic calls before 1 p. m.

THE BUFFALO MEDICAL JOURNAL will publish regularly. full transactions of any professional organization in western New York at cost. Personal notices, brief reports of society proceedings, and the like, are always welcome and will be published without charge.

Subscriptions may commence at any time. $2.00 per year in advance,

VOL. LXVIII. AUGUST, 1912 No. 1

*It is wickeder to be idle on week days than to work on Sundays.*

### The Past Year and the Future

It is more than a year since the present editor began, informally, to assist in the conduct of this JOURNAL, a little less than a year since he assumed full charge—reluctantly, from apprehension of interference with more practical and more necessary work. Thanks to the unanimous and cordial support of the profession, this apprehension has proved to be unfounded and, with increasing familiarity with details and improved assistance, the additional labor will probably prove less exacting. With allowance for subscription sharing, the subscription list for Buffalo and neighboring towns is estimated to be within about 80 of a possible maximum. For Rochester, we need about 50 more subscriptions, and the same number for the towns in that zone of our territory. To the east and south, the field for extension increases as the radius lengthens. The appointment of Associates conveniently located has proved to be wise from every standpoint, and local centers will probably be added as the need arises. While the JOURNAL aims especially to serve western New York as a medium for exchange of news, lines of political geography are arbitrary and we have a considerable circulation in Ontario, Pennsylvania and Ohio, following established lines of travel. With no special effort toward a general circulation, we also mail to almost all states and countries of the globe, and this circulation is gradually increasing, almost every day's mail including requests for samples or comments showing that the work of our contributors is appreciated, and that we reach, indirectly, localities that we do not realize.

We wish to impress on our readers the importance of putting forth their best literary efforts in the field of their practice and upon our readers throughout our territory that we wish to serve all parts of it equally, in all ways. The JOURNAL is the third oldest in this continent, it was original called the BUFFALO MEDICAL JOURNAL and, with some slight variations, has always borne that name and it is printed in Buffalo, so that it seems inadvisable to change the name, if indeed, any short designation could be found to indicate its rather broad local scope. Figures speak eloquently and this is our apology for impressing on our readers in a rather sordid way, the debt they owe the advertisers, in the way of careful attention to the advertisements. Each copy mailed to a Buffalo subscriber costs $2.05 for printing and mailing alone; each copy to a neighboring town about 1.90—providing that payments are made without requiring bills and receipts and without exchange. After passing the first thousand of the profession, outside of Buffalo and neighboring counties, a small gross profit exists but, at the maximum estimate of circulation, not sufficient to publish a journal of the same size without advertisements, unless a very broad and large field could be covered by an entire change in policy and scope.

Notwithstanding, the JOURNAL inforces, so far as possible, the highest standard of advertising ethics, including those of simple honesty—as the fulfillment of a contract, however conflicting with personal opinion—fair play, and common sense. In the last ten months, enough advertising has been refused to pay the expense of more than one issue. We invite suggestions on this, as well as other points.

Scientific material of the highest grade is available among our own subscribers but we shall, from time to time, include articles from distant parts, especially Europe. Several of the most important advances in professional achievement and policy, especially along educational and legislative lines, have originated in western New York. At least three such movements have started within the last year and a half, have been first published in this JOURNAL, and have set up sympathatic vibrations throughout the profession of the country.

